# Pharmacokinetic, Antiproliferative and Apoptotic Effects of Phenolic Acids in Human Colon Adenocarcinoma Cells Using In Vitro and In Silico Approaches

**DOI:** 10.3390/molecules23102569

**Published:** 2018-10-08

**Authors:** Lana de Souza Rosa, Nathállia Araújo Jordão, Nathália da Costa Pereira Soares, Joelma Freire de Mesquita, Mariana Monteiro, Anderson Junger Teodoro

**Affiliations:** 1 Laboratory of Functional Foods, Food and Nutrition Program, UNIRIO, Rio de Janeiro 22290-240, Brazil; lanasrosa@gmail.com (L.d.S.R.); nathalliasomerhalder@gmail.com (N.A.J.); nathy.soares@gmail.com (N.d.C.P.S.); 2 Laboratory of Bioinformatics and Computational Biology, Program Postgraduate in Molecular and Cellular Biology, UNIRIO, Rio de Janeiro 22290-240, Brazil; jomesquita@gmail.com; 3 Nutrition Program, Laboratory of Functional Foods, UFRJ, Rio de Janeiro 22290-240, Brazil; macostamonteiro@gmail.com; 4 Department of Food Science, Food and Nutrition Program, Federal University of Rio de Janeiro State, Rio de Janeiro. Av. Pasteur, 296-Urca, Rio de Janeiro-RJ 22290-240, Brazil

**Keywords:** phenolic acid, colon cancer, cell cytotoxicity

## Abstract

Colon cancer is the second most common cause of cancer deaths in the USA and Europe. Despite aggressive therapies, many tumors are resistant to current treatment protocols and epidemiological data suggest that diet is a major factor in the etiology of colon cancer. This study aimed to evaluate the antioxidant activity and the influence of 3,4-dihydroxyphenylacetic (3,4-DHPAA), *p*-coumaric (*p*-CoA), vanillic (VA) and ferulic (FA) acids on cell viability, cell cycle progression, and rate of apoptosis in human colon adenocarcinoma cells (HT-29). The results showed that all compounds tested reduce cell viability in human colon cancer cells. 3,4-DHPAA promoted the highest effect antiproliferative with an increase in the percentage of cells in G_0_/G_1_ phase, accompanied by a reduction of cells in G_2_/M phase. Cell cycle analysis of VA and FA showed a decrease in the proportion of cells in G_0/_G_1_ phase (10.0 µM and 100.0 µM). *p*-CoA and FA acids increased the percentage of apoptotic cells and non-apoptotic cells. 3,4-DHPAA seems to be the substance with the greatest potential for in vivo studies, opening thus a series of perspectives on the use of these compounds in the prevention and treatment of colon cancer.

## 1. Introduction

Colon cancer is the second most prevalent malignancy and the third leading cause of cancer-related mortalities worldwide, resulting in ~500,000 mortalities every year [1]. Approximately, 1 million new cases are diagnosed every year [2]. In general, colon cancer is related to environmental factors, mainly dietary, genetic predisposition and obesity, among other factors [3]. Colon cancer develops through a multistage process that could be recognized at a histopathological level by progression from mucosa to invasive carcinoma [4].

Epidemiological data suggest that diet is a major factor in the etiology of colon cancer. High consumption of red meat, animal fat, alcohol and refined cereals is linked to higher incidence of these cancers in Western societies, whereas protective effects of fruits, vegetables and whole grains have been suggested [5].

A wide variety of fruits and vegetables provides a range of nutrients and different bioactive compounds including phytochemicals (phenolics, flavonoids, and carotenoids), vitamins (vitamin C, folate, and pro-vitamin A), minerals (potassium, calcium, and magnesium), and fibers [6]. Phenolic compounds have been reported to possess important biological properties such as anticancer, antiviral, antioxidant and anti-inflammatory activities [7]. Phenolics are compounds possessing one or more aromatic rings with one or more hydroxyl groups and generally. It has been generally classified as subgroups of phenolic acids, flavonoids, stilbenes, coumarins, and tannins [6].

Ferulic acid (FA) and *para*-coumaric acid (*p*-CoA), members of the hydroxycinnamic acid family, are the main phenolic compounds in cereal bran, one of the major fiber sources in a healthy diet [8]. *p*-CoA is the most abundant isomer of cinnamic acid, widely found in edible plants such as peanuts, tomatoes, carrots. *p*-CoA is reported to have antitumor and anti-mutagenic activities [9]. Vanillic acid, a benzoic acid derivative, is an oxidized form of vanillin produced during the conversion of vanillin into ferulic acid. Vanillic acid is one of the major components of “natural vanilla” aroma and it is used as a flavoring agent. It has also an antioxidant and antimicrobial activity and therefore it can be considered as a potential food preservative [10,11]. Colonic bacteria can convert flavonoids into large number of simple phenolic acids that can be absorbed into the circulation, and exert biological effects in the body. Previous publication indicated that quercitin is metabolized to 3-hydroxyphenylacetic acid, 3,4-dihydroxybenzoic acid. 3,4-dihydroxyphenylacetic acid, whose structure is analogous to that of caffeic acid, but it lacks a double bond in the aliphatic chain. It also exerts anti-oxidant properties, antiplatelet activity and anxiolytic activity [12,13].

A range of evidence supports the theory of anticancer properties of phenolic acids, although the mechanisms are still not fully understood, but may include scavenging free radicals, induction of enzymes involved in the metabolism of xenobiotics, regulation of gene expression, modulation of cellular signaling pathways including those involved in DNA damage repair, cell proliferation, apoptosis and invasion [14]. Phenolic acids have been reported to inhibit transcription factors linked to inflammation, pro-inflammatory cytokines, COX-2, lipoxygenases (LOX), inducible nitric oxide synthase (iNOS) [14]. Therefore, this study aimed to evaluate the antioxidant activity and the influence of 3,4-dihydroxyphenylacetic (3,4-DHPAA), *p*-coumaric (*p*-CoA), vanillic (VA) and ferulic (FA) acids on cell viability, cell cycle progression, and rate of apoptosis in human colon adenocarcinoma cells (HT-29).

## 2. Results

### 2.1. Antioxidant Activity and In Silico Approach of 3,4-DHPAA, p-CoA, VA and FA

All compounds were analyzed by the DPPH method at concentrations below 25.0 μM (Table 1), where it is observed that there was no significant reduction of the DPPH radical (results not shown). After 30 min of testing, the percent reduction of DPPH tended to stabilize at the concentration of 25.0 μM. It was observed in the same method, that the 3,4-DHPAA presented the highest percentage of reduction of the radical, followed by VA, FA and *p*-CoA.

The ORAC assay (Table 1) revealed that *p*-CoA and 3,4-DHPAA showed the highest antioxidant, followed by VA and FA. 3,4-DHPAA and VA showed higher antioxidant activity compared to FA and *p*-CoA by FRAP assay, corroborating with the results observed in the DPPH assay. All compounds were also shown to be significantly different at all concentrations studied (*p* ≤ 0.05). In the ABTS assay (Table 1), 3,4-DHPAA showed the highest antioxidant activity, as well as in the DPPH and FRAP assay.

The predictive results (Table 1) presented by pkCSM and LAZAR algorithms were compared and presented in Table 1. Both predictive strategies suggested 3,4-DHPAA, *p*-CoA and VA were non-mutagenic in the Ames toxicity test, although the predictive results using the in silico approach indicated the absence of mutagenicity in Ames test, in FA. pkCSM and LAZAR prediction did not suggest a carcinogenic effect of any of the compounds in mice and rats separately. However, VA and FA had suggested carcinogenic effect in rats by pkCSM and LAZAR prediction. The maximum tolerated dose in humans were predicted in the same range using both strategies.

### 2.2. Effect of 3,4-DHPAA, p-CoA, VA and FA in Cell Viability

**Figure 1:**
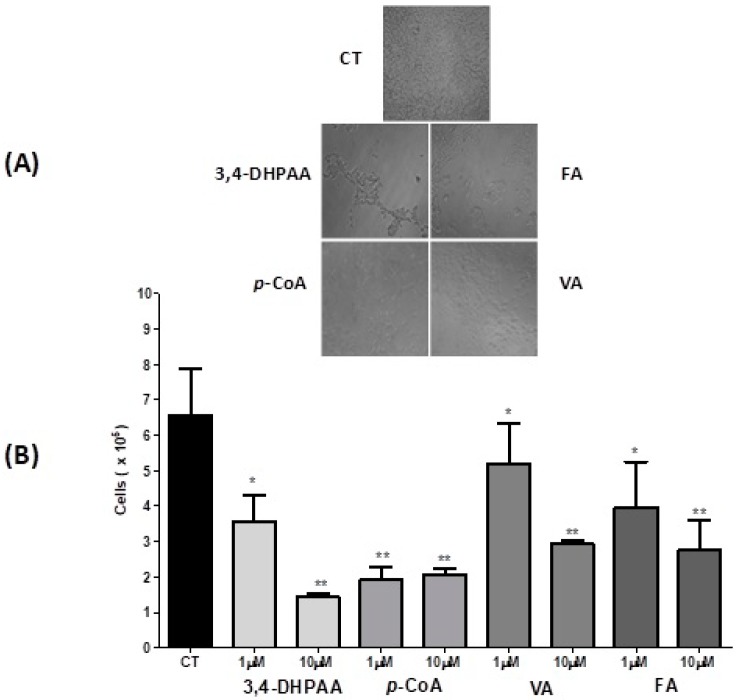
Effect of 3,4-DHPAA, *p*-CoA, VA and FA (100 μM) on HT-29 cells 24 h after incubation (**A**). Effect of 3,4-DHPAA, *p*-CoA, VA and FA on cell viability (mean ± SD) (HT-29) 24 h after incubation (**B**). Significant differences between the untreated cells (CT) and those incubated with the respective acids (1.0 μM and 10.0 μM) were compared by the one-way ANOVA test, with Tukey post-test (* *p* < 0.05; ** *p* < 0.01).

The dye exclusion test is used to determine the number of viable cells present in a cell suspension. It is based on the principle that live cells possess intact cell membranes that exclude certain dyes, such as Trypan Blue. In this test, a cell suspension is mixed merely with dye and then visually examined to determine whether cells take up or exclude dye. Cells treated with 3,4-DHPAA, *p*-CoA, VA and FA presented a percentage reduction in cell viability at 1.0 μM with a significant difference (*p* < 0.05) when compared to control cells. At the concentration of 10.0 μM the cells treated with 3,4-DHPAA presented the highest percentage of reduction of cell viability, followed by *p*-CoA, FA and VA with significant difference (*p* < 0.01) when compared to the control group (Figure 1).

**Figure 2:**
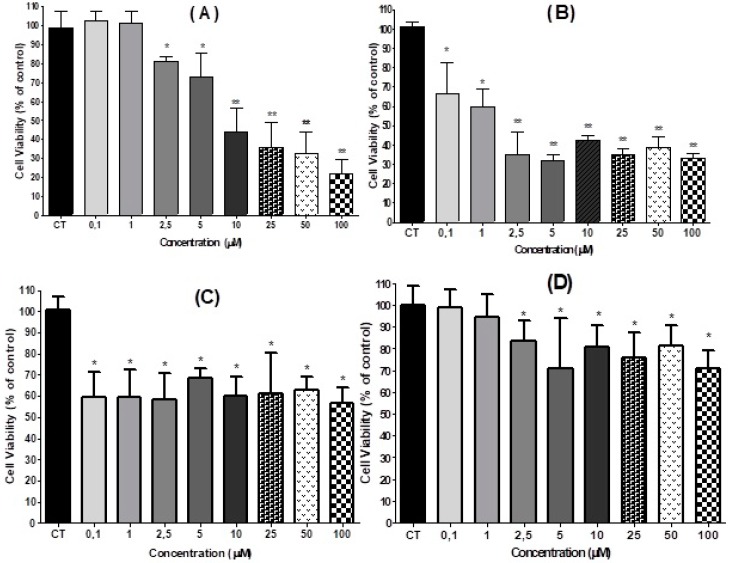
Effect of 3,4-DHPAA (**A**), FA (**B**), *p*-CoA (**C**) and VA (**D**) on viability (mean ± SD) of HT-29 cells 24 h after incubation. Significant differences between the untreated cells (CT) and those incubated with the respective acids (0.1 μM to 100 μM) were compared by the One-way ANOVA test, with Tukey post-test (* *p* < 0.05; ** *p*< 0.01).

HT-29 cells were seeded and after recovery for 24 h, cells were incubated with 0.1 µM to 100.0 µM of 3,4-DHPAA, *p*-CoA, VA and FA during 24 h. We used the MTT assay to monitor the cell viability. 3,4-DHPAA did not change the cellular growth profile at concentrations of 0.1 µM and 1.0 µM. A more significant dose-dependent decrease (66.0%) was identified in cells treated with concentrations higher than or equal to 10.0 µM of 3,4-DHPAA (Figure 2A). FA showed an inhibitory growth effect of viable cells when compared to control after treatment with all concentrations tested. FA promoted on average inhibition of 63.0% of cells treated with 2.5 µM to 100.0 µM (Figure 2B) When HT-29 cells were incubated with *p*-CoA, a significant reduction of HT-29 viable cells was observed, with an average reduction of 40.0% and without any significant difference between these concentrations (*p* > 0.05, Figure 2C). No effect was shown after VA treatment with lower doses (0.1 µM to 1.0 µM), however, high concentrations of VA (2.5 µM to 100.0 µM) inhibited cell viability with an average inhibition of 23% without difference among the concentrations tested (*p* > 0.05) (Figure 2D).

### 2.3. Effect of 3,4-DHPAA, p-CoA, VA and FA in Cell Cycle Progression

**Figure 3:**
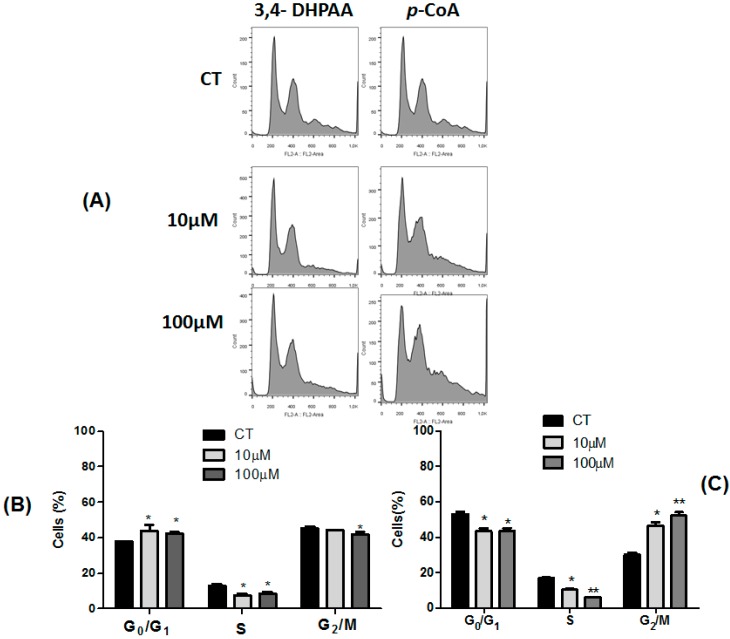
Effect of 3,4-DHPAA and *p*-CoA on cell cycle progression in HT-29 cells 24 h after incubation. The phases of the cell cycle are illustrated at control (CT) and treated with 10.0 μM and 100.0 μM of these compounds in figure (**A**). The quantitative results of the effect of 3,4-DHPAA compound on this cell line are shown in figure (**B**) and *p*-CoA in figure (**C**). The experiment is expressed as mean ± SD. Significant differences between untreated and treated (10 μM and 100 μM) cells were compared by the One-way ANOVA test, with Tukey posttest (* *p* < 0.05; ** *p* < 0.01).

To monitor the influence of 3,4-DHPAA, *p*-CoA, VA and FA on the cell cycle, the cells were incubated with 10.0 μM and 100.0 μM concentrations of each compound for 24 h and the percentage of viable cells in different cell cycle phases were quantified. In HT-29 cells, 3,4-DHPAA (10.0 μM and 100.0 μM) induced an increase in the percentage of cells in G_0_/G_1_ phase followed by a decreased cell number in S phase (*p* < 0.05) (Figure 3A,B).

3,4-DHPAA (100 µM) treatment also promoted a decrease of cells in G_2_/M phase when compared to the control group (*p* < 0.05). *p*-CoA (10.0 μM and 100.0 μM) promoted a significant decrease in G_0_/G_1_ and S phases, followed by a cell cycle arrest in G_2_/M phase when compared to untreated cells (Figure 3A,C).

**Figure 4:**
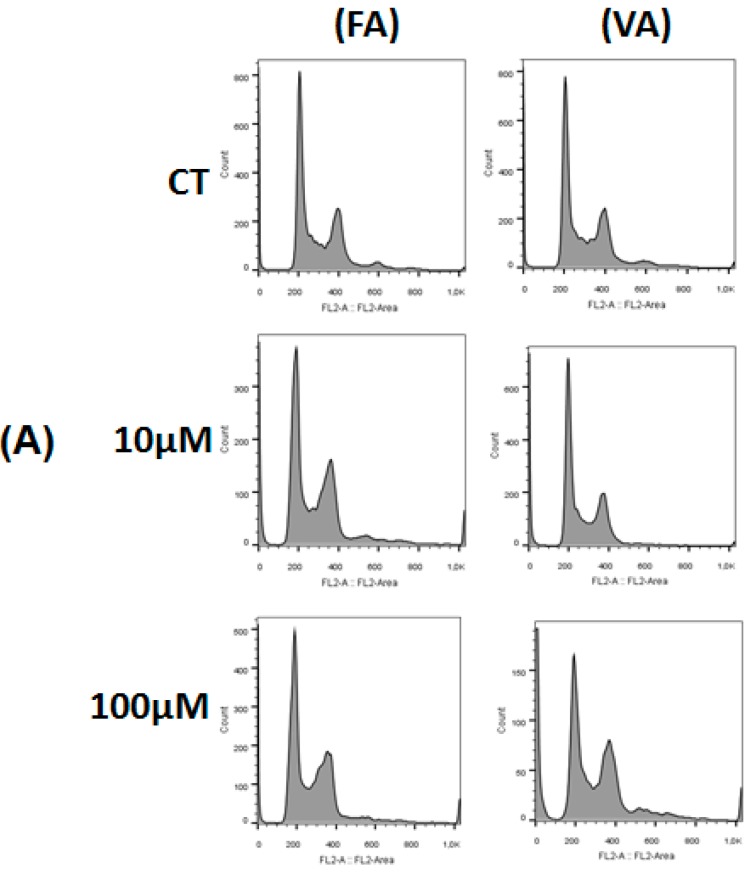

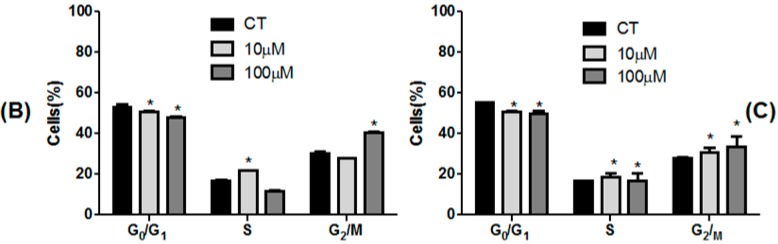
Effect of FA and VA on cell cycle progression in HT-29 cells 24 h after incubation. The phases of the cell cycle are illustrated at control (CT) and treated with 10 μM and 100 μM of these compounds in figure (**A**). The quantitative results of the effect of FA compound on this cell line are shown in figure (**B**) and VA in figure (**C**). The experiment is expressed as mean ± SD. Significant differences between untreated and treated (10 μM and 100 μM) cells were compared by the One-way ANOVA test, with Tukey posttest (* *p* < 0.05).

A decrease in the percentage of HT-29 cells in G_0_/G_1_ phase was observed after FA (10 µM and 100 µM) incubation. A reduction of cells in S phase and a higher percentage of cells in the G_2_/M phase was noted after treatment with FA at 10.0 μM and 100.0 μM, respectively (Figure 4A,B). VA (10 µM and 100 µM) induced a decreased in the percentage of cells in G_0_/G_1_ phase, followed by an increase of cell number in S and G_2_/M phases when compared to control group (*p* < 0.05, Figure 4A,C).

### 2.4. Effect of 3,4-DHPAA, p-CoA, VA and FA in Apoptosis

**Figure 5:**
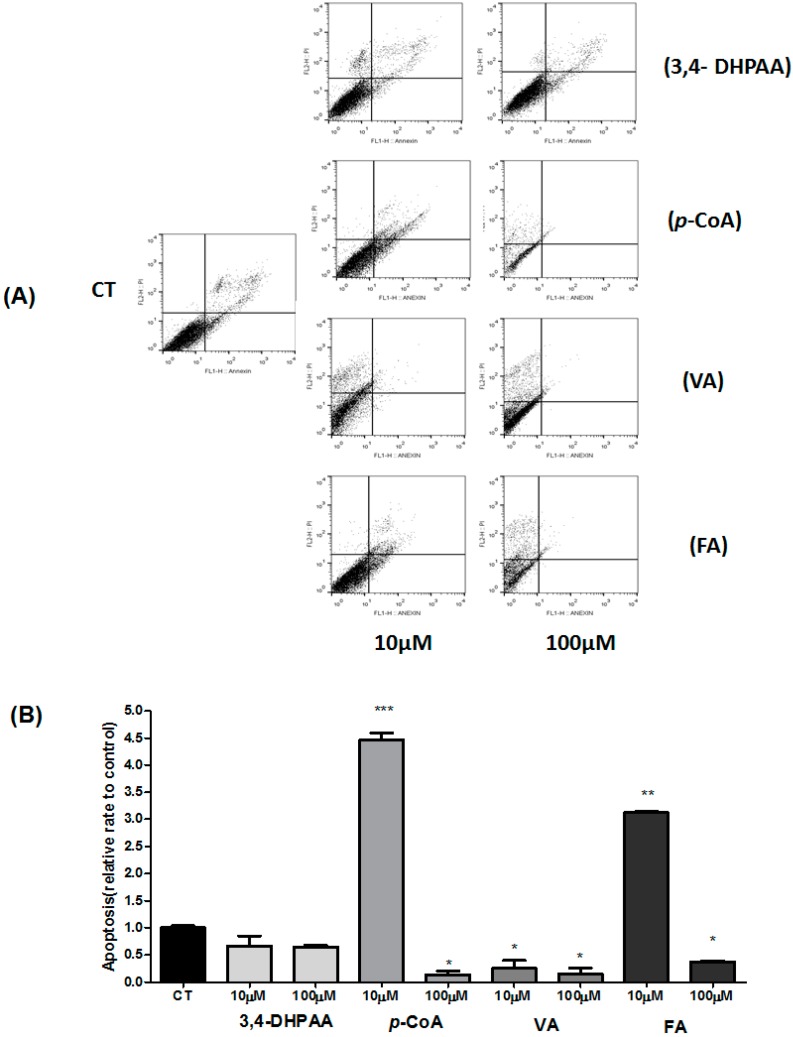
Effect of 3,4-DHPAA, *p*-CoA, FA and VA on rate of apoptosis in HT-29 cells after 24 h after incubation. Flow cytometry analysis of 3,4-DHPAA, *p*-CoA, FA and VA are illustrated in (**A**) according to the exposure time and concentration of these compounds. (**B**) The quantitative effects of 3,4-DHPAA, *p*-CoA, FA and VA on HT-29 cells are after 24 h exposure. The experiment is expressed as mean ± SD, with significant differences between untreated cells (CT) and treated (10.0–100.0 µM) compared by ANOVA followed by Tukey post-hoc test. * *p* < 0.05.

We examined next the effect of 3,4-DHPAA, *p*-CoA, VA and FA on different stages of the HT-29 cell death process for 24 h. Table 2 shows the percentages of viable, early apoptotic, late apoptotic, and non-apoptotic cells after treatment with 3,4-DHPAA, *p*-CoA, VA and FA (10.0 μM and 100.0 μM) and Figure 5 shows the influence of 3,4-DHPAA, *p*-CoA, VA and FA on the apoptosis rate. A significant decrease (*p <* 0.05) in the percentages of viable cells (10.0 μM and 100.0 μM) and significant increase (*p <* 0.05) in the percentages of apoptotic cells (100.0 µM) was observed after treatment with 3,4-DHPAA compared to untreated cells (control group). The percentage of non-apoptotic cells showed an increase (*p <* 0.05) after treatment with 3,4-DHPAA (10.0 μM and 100.0 μM, Table 2).

After treatment with *p*-CoA (10.0 µM), HT-29 cells showed a decrease in the population of viable cells (*p <* 0.05) and an increase of apoptotic cells (early and late apoptotic cells) compared to control group. The percentage of viable cells did not change significantly (*p >* 0.05) after treatment with VA (10.0 µM and 100.0 µM) compared to untreated cells. However, a significant difference in the percentage of cells in apoptosis (early and late apoptotic cells), compared to control (Table 2) was observed. After treatment with FA ((10.0 µM and 100.0 µM), HT-29 cells showed a decrease in the population of viable cells (*p* < 0.05) compared to control group. Also, when they were incubated with *p*-CoA (100.0 μM), VA ((10.0 µM and 100.0 µM) and FA (100.0 μM), the HT-29 cells showed an enhanced number of non-apoptotic cells, indicating a probable toxic effect at high doses of these compounds (Table 2 and Figure 5).

Relative apoptosis rate data show that only *p*-CoA and FA acids promoted increased apoptosis after incubation with the lower dose (10.0 µM). No effect (*p* > 0.05) was observed after treatment with 3,4-DHPAA and VA (10.0 µM and 100.0 µM, Figure 5).

## 3. Discussion

Some naturally occurring phenolic acids and analogs are known to display a wide variety of biological functions, in addition to their primary antioxidant activity, which is mainly related to modulation of carcinogenesis. Indeed, many phenolic compounds have been investigated for their potential use as cancer chemopreventive agents [15]. The results of the present study provide supporting evidence supporting the role of 3,4-DHPAA, *p*-CoA, VA and FA in the prevention and treatment of colon cancer.

In our study, the antioxidant activity of 3,4-DHPAA, *p*-CoA, VA and FA was analyzed by four different methods. For the four methods used, the high antioxidant activity of the compounds was observed. It was observed in our study that 3,4-DHPAA showed to have an antioxidant activity superior to the other phenolic acids by DPPH, FRAP and ABTS assays.

Monagas et al. [16] observed that 3,4-DHPAA derived from microbial phenolic metabolites inhibited the secretion of pro-inflammatory cytokines (TNF-α, IL-1b and IL-6) in lipopolysaccharide-stimulated peripheral blood mononuclear cells in healthy volunteers. These results indicate that dihydroxylated phenolic acids derived from microbial metabolism present marked anti-inflammatory properties, providing additional information about the health benefits of dietary polyphenols and their potential value as therapeutic agents.

Antioxidant assays (FRAP and DPPH) demonstrated that VA presented the second highest antioxidant potential. In the ABTS and ORAC assay, VA presented a lower antioxidant activity, when compared to the other compounds. The results found in our study are similar to those reported by Tai et al. [17]. When analyzing the antioxidant activity of VA by DPPH, ORAC and ABTS Tai et al. [17] observed that VA was able to repress the three radicals. In the ORAC assay, VA showed a partial inhibition over time in the fluorescence decay profile and a significant depletion of the ABTS radical.

Kiliç and Yesiloglu [18], showed in their study that *p*-CoA had an effective DPPH scavenging, ABTS scavenging and ferric ions (Fe^3+^) reducing power, suggesting that *p*-CoA can be used in the pharmacological and food industry because of these properties. In our results, we can observe that *p*-CoA showed high antioxidant activity by the ORAC method and a lower activity by the other methods FA demonstrated a high antioxidant activity by ABTS assay. The FA provides hydrogen for the neutralization of the free radicals combated, which gives it antioxidant effect.

A range of analytical methods for the determination of antioxidant properties of phenolic compounds is available. Our work demonstrates that different assay methods differ from one another regarding reaction mechanisms, oxidant species, reaction conditions and the way the final results were expressed. A number of antioxidant assays, such as DPPH, ABTS, ORAC, FRAP and TEAC, are commonly used. In a few cases, biological assays, typically involving cell culture, also provide useful information on the effectiveness of antioxidants. The methods used in our study to evaluate the antioxidant activity of these compounds were satisfactory and adequate. These methods are important because free radicals and reactive oxygen species (ROS) have been implicated in contributing to the processes of aging and disease. Our data confirmed what is already described in the literature regarding the high antioxidant activity of 3,4-DHPAA, *p*-CoA, VA and FA.

Cancer is characterized by deregulation of apoptosis, cell proliferation, invasion, angiogenesis and metastasis. It is desirable that there be a compound capable of inhibiting the proliferation of cancer cells. Cell viability inhibition by 3,4-DHPAA, *p*-CoA, VA and FA has already been described in other studies. Gao et al. [19] studied the effects of phenolic acid products formed during colon digestion from green tea, black tea, citrus fruit with rutin or soy digested under the same conditions using pooled human colonic microbiota. Only 3,4-DHPAA exhibited antiproliferative activity in prostate and colon cancer cells. 3,4-DHPAA was significantly (*p* < 0.005) more inhibitory in colon cancer cells (HCT116) compared with an immortalized normal intestinal epithelial cell line (IEC6) with IC_50_ 90 µmol/L. The antiproliferative activity of 3,4-DHPAA may be due to its catechol structure [20].

Henning et al. [21] found similar results which reported that the plasma concentration of 3,4-DHPAA increased after the black tea intake when tested in vitro. It has been described that 3,4-DHPAA exercise antiproliferative activity in colon cancer cells (HCT116). Our results demonstrated that 3,4-DHPAA promoted a significant reduction in the percentage of viable cells, around 66% at concentrations equal to or higher than 10 μM after 24 h incubation.

FA promoted a significant reduction (35.0%) in cell viability after treatment with concentrations of 0.1 and 1.0 μM. Likewise, a higher reduction (63.0%) was observed at concentrations equal to or greater than 5.0 μM. Previous studies using FA demonstrated an inhibitory effect on cell viability on colon cancer cells [22]. This effect was confirmed by in vivo tests in rats by Hudson et al. [23]. The potential health benefits of ferulic acid and other hydroxycinnamic acids have been related mostly to their effective antioxidant activity. Hydroxycinnamic acids can protect low-density lipoprotein (LDL) from oxidative modifications and thereby reduce atherogenesis. They also exhibit inhibitory effects on tumor promotion and can block the formation of mutagenic compounds such as nitrosamines [24].

Janicke et al. [25] showed that FA and *p*-CoA inhibit the proliferation of Caco-2 cells (43–75%) after 2–3 days of treatment. These data are in agreement with Hudson et al. [23], where SW480 cells (colon cancer cells) treated with FA had reduced cell viability. Ferguson et al. [26] have confirmed antioxidant effects of hydroxycinnamic acids and extended their range of known activities to include protection against DNA and chromosome breakage, as well as modulation of the activity of specific enzymes previously associated with carcinogenesis.

Among the compounds studied, VA was the one that presented the lowest percent reduction in viability of HT-29 cells, an average percentage of 23%. Ho et al. [27] in their results of cell viability assay, showed that vanillin exerts more cytolytic and cytostatic effect on HT-29 cells, while vanillic acid did not show any cytolytic or cytostatic effect at a concentration up to 500 µg/mL.

Our results in MTT assay corroborate with the data obtained in cell counting with Neubauer chamber, where the compounds had the following order in the reduction of cell viability: 3,4-DHPAA > FA > *p*-CoA > VA. The data available in the literature and those found in our study suggest that mainly 3,4-DHPAA and FA may play a role as an inhibitor of cell viability and prevent the proliferation of human colon cancer cells.

Some authors have already studied the cytotoxicity of phenolic acids in normal and colon cancer cells and revealed that extracts of barley (*Hordeum vulgare* L.) as a rich source of different bioactive compounds inhibited the proliferation of cancer cells. The NR (neutral red) method study confirmed the low cytotoxic activity of the tested extracts to normal human colon epithelial cells (CCD 841 CoTr). The obtained results indicate a cancer chemopreventive potential of in colon carcinoma [28]. Subramanian et al. [29] showed that gallic acid (GA) treatment was non-toxic to normal cells. These results indicate that the phenolic compound GA has insignificant inhibitory activity against colon cancer cells even at a very high concentration. These data reinforce the results found by our work in silico analysis that demonstrated low toxicity of these compounds already described in animal models, with the exception of VA and FA.

Deregulated cell-cycle control is a fundamental aspect of cancer. Normal cells only proliferate in response to developmental or other mitogenic signals that indicate a requirement for tissue growth, whereas the proliferation of cancer cells proceeds essentially unchecked. Therefore, cancer cells proliferate because of defects in internal and external proliferation-inhibitory signals [30]. A limiting step in the cell cycle, which often is not regulated in cancer, is the progression of cells in the first stage (G_1_) of the cycle to the S phase [31].

In Caco-2 cells, FA and *p*-CoA decreased the proportion of cells in the G_1_ phase and increased the proportion of cells in the S and G_2_ phases. Treatment with FA significantly increased the length of the S phase, while *p*-CoA did no. It was concluded that FA and *p*-CoA inhibited cell proliferation by presumably affecting different cell cycle phases, and this warrants further investigations because this inhibition may be one explanation for the diet-related protection against cancer [25].

In our study, cell cycle revealed that 3,4-DHPAA (10 µM and 100 µM) was able to induce an increase in the percentage of HT-29 cells in G_0_/G_1_ phase and decrease cells in S and G_2_/M phases after 24 h of treatment compared to control. These data suggest cell cycle arrest in G_0_/G_1_ phase, where the cells are unable to reach the stage of mitosis and multiply. In this context, 3,4-DHPAA demonstrated an important effect on modulating cell cycle.

In this work, the cell cycle analysis of the VA and FA showed a decrease in the proportion of cells in the G_0_/G_1_ phase (10 μM and 100 μM) 24 h after incubation. Also, VA did not promote an increase in the percentage of apoptotic cells (early and late apoptotic cells). Our data also revealed that FA decreased the percentage of viable cells (10 μM), promoted an elongation S phase and decreased G_2_/M phase of the cell cycle, with an increase in the percentage of apoptotic cells (early and late apoptotic cells). At the concentration of 100 μM, this percentage of apoptotic cells was reduced and increased by non-apoptotic cells.

The understanding of the apoptotic mechanisms allows the development of new strategies in the treatment of cancer. Such strategies are based on the induction of death in tumor cells and a higher response to treatments with radiation and cytotoxic agents [32]. Apoptosis is the endpoint of at least one of the differentiation pathways of colon epithelial cells, a process that results in apoptotic cell death should also minimize the proliferative signal. In fact, in our model, the increase in apoptosis cell death rate after 24 h of incubation with *p*-CoA with an increase of cells in the G_2_/M phase of the cell cycle.

*p*-CoA and FA acids increased the relative apoptosis rate by 4.40 and 3.12 times, respectively, at the lowest concentrations tested. At high concentrations of *p*-CoA, FA and VA were able to increase the percentage of non-apoptotic cells, thus indicating a toxic effect of the high concentrations of these compounds. Previous studies related different molecular mechanisms of phenolic acids in apoptosis. VA significantly up regulated the expression and protein level of Procaspase-3, Procaspase-9, Bax and p53 when compared to control cells [33]. FA could inhibit the expression and activity of cytotoxic enzymes, including inducible nitric oxide synthase, caspases and cyclooxygenase-2 [34] and modulation of p53, Bax, and GADD45 [35]. Other authors hypothesize that the increased ROS generation may result in the activation of p53 in the p-CoA treated colon cancer cells. This may in-turn would have caused the up-regulation of Bax and downregulation of Bcl2 which are the down-stream targets of p53 resulting in apoptosis [9]. In PC-12 cells, the combination of 3,4-dihydroxyphenylacetic acid and nitric oxide induced an early mitochondrial membrane potential dissipation accompanied by the release of cytochrome c in both cases. Nitric oxide individually and in combination with 3,4-dihydroxyphenylacetic acid activate caspase-3 and caspase-9 [36]. It is likely that the pro-apoptotic effects promoted by phenolic acids in this work may be derived from the activation of caspases and Bax and modulation p53, already reported in other reports, however, but more studies are necessary to identify the mechanisms involved.

In vivo studies have also explored the anti-tumor activities of *p*-CoA and its conjugates. In a mouse tumor model induced by injection of RENCA cells, intraperitoneal administration of *p*-CoA for 1 week significantly decreased tumor volumes (by 53%) and weights (by 40%) by reducing angiogenesis within a tumor [37]. *p*-CoA also exerts moderate protection against 1,2-dimethylhydrazine-induced colon cancer in rats [38]. Cancerous rats fed with a high-fat diet containing 0.1% *p*-CoA exhibited a decrease in colorectal carcinomas/rat from 2.5 to 2.0, the mean number of intestinal tumors/rat from 5.5 to 4.7, and the total number of tumors in the small intestine from 0.87 to 0.45 tumors/rat [39].

Vanillic acid has been reported to exert a suppressive effect on the concanavalin A-induced liver injury due to their ROS (generated by activated NADPH oxidase in the lymphocytes) scavenging action. There is evidence that this compound significantly decreases the transaminase activity and suppress the disorganization of the hepatic sinusoids; they scavenge the ROS to suppress hepatocyte death. They also suppress the production of inflammatory cytokines, tumor necrosis factor (TNF)-α, and interferon (IFN)-γ in mice having liver inflammation. This phenolic compound is also found to dramatically suppress liver fibrogenesis in the chronic CCl4-treatment model [40].

According to Mancuso et al. [41], FA has a low degree of toxicity after oral administration. Some authors in their studies observed a reduction in mobility, piloerection and lacrimation in F344 rats treated with FA; in the case of single administration of FA higher than 1929 mg/kg, the death of rats occurred during the first 24 h of the 14-day period of observation [42]. By contrast, death from gastrointestinal bleeding occurred 24 h from the administration. Finally, LD50s equal to 2445 mg/kg and 2113 mg/kg were calculated for male and female rats, respectively [43], whereas an acute LD50 of 3200 mg/kg was calculated in mice [44]. This low toxicity has been confirmed by numerous experimental studies [41].

Thus, although the in silico analysis shows low toxicity for these phenolic substances, the results of the in vitro analysis suggest that 3,4-DPHHA exercise the higher antiproliferative effect with low toxicity. The daily uptake of 3,4-DPHHA with food is 7.27 mg, and it is present in nanomolar concentrations in human plasma, so it is opening new perspectives for studies of this substance in vivo models for clinical implications.

## 4. Materials and Methods

### 4.1. Standards and Chemicals

Dulbecco’s cell culture medium, bovine serum albumin and 3,4-DHPAA, *p*-CoA, VA and FA were purchased from Sigma-Aldrich Chemical Company (St. Louis, MO, USA). Fetal bovine serum was purchased from Laborclin (Campinas, São Paulo, Brazil). Tissue culture flasks and cell scrapers were purchased from Nunc (Roskilde, Denmark). All the chemicals were of analytical grade.

### 4.2. Antioxidant Activity Analyses

#### 4.2.1. Oxygen-Radical Absorbance Capacity Assay (ORAC)

The ORAC procedure used an automated plate reader (SpectraMax i3x, Molecular Devices, San Jose, CA, USA) with 96 well plates [45,46]. Experiments were conducted in phosphate buffer pH 7.4 at 37 °C. Peroxyl radical was generated using 2,2’-azobis (2-amidino-propane) dihydrochloride which was prepared fresh for each run. Fluorescein was used as the substrate. Fluorescence conditions were as follows: excitation at 485 nm and emission at 520 nm. The standard curve was linear between 0 and 50 mM Trolox. Results are expressed as mEq Trolox/L.

#### 4.2.2. Ferric Reducing Ability (FRAP)

The extracts were measured for antioxidant activity by FRAP according to Bauer et al. [47]. Aliquots of 2.7 mL of TPTZ reagent (ferric 2,4,6-tripyridyl-s-triazine) were mixed with 0.5 mL of sample. After 30 min at 37 °C temperature, the absorbance was read at 595 nm. The antioxidant capacity (FRAP) was expressed as Fe^3+^ equivalents (μmol ferrous sulfate/µmol of compound).

#### 4.2.3. DPPH Assay

Aliquots of 0.5 mL of the extracts were mixed with 2.5 mL DPPH methanolic solution (0.06 mM) and allowed to react for half an hour, in the dark. Measurements were performed at 515 nm applying a Turner^®^ 340 spectrophotometer (Barnstead/Thermolyne, Dubuque, IA, USA). The analysis was performed in triplicates and the decline in the DPPH radical absorbance concentration caused by the samples was measured. The results expressed as the percentage reduction of the DPPH [48].

#### 4.2.4. Trolox Equivalent Antioxidant Capacity (ABTS/TEAC)

The TEAC^+^ cation was prepared by mixing a TEAC stock solution (7 mM in water) with 2.45 mM potassium persulphate. This mixture was allowed to stand for 16 h at room temperature until the reaction was completed and the absorbance was stable. The antioxidant capacity assay was carried out by the improved ABTS/TEAC method of Bauer et al. [47]. TEAC solution (2.5 mL) was added to samples or commercial antioxidant (Trolox) and mixed thoroughly. Absorbance was recorded at 734 nm for 6 min. Results were calculated as µmol Trolox/µmol and µmol ascorbic acid/µmol.

### 4.3. Cell Culture and Treatment Protocol

Human colon adenocarcinoma cell line (HT-29) was obtained and certified from the Rio de Janeiro Cell Bank (Inmetro, Rio de Janeiro, Brazil) that have certified their identity and quality. HT-29 cells were plated in 25 cm^2^ tissue culture flasks, 5.0 × 10^6^ cells/flask, and maintained routinely in Dulbecco’s medium supplemented (DMEM) with 10% fetal bovine serum and 2 g/L HEPES buffer, pH = 7.4, under 5% CO_2_ atmosphere. Cells were passaged at 70–80% confluence, about twice a week by trypsinization. Cells were seeded at 2.0 × 10^4^ cells/cm^2^ in 6-multiwell plates (2 mL of standard culture medium) for Cell Counting with Neubauer Chamber, cell cycle progression, and apoptosis analyses and in 96-multiwell plates (200 mL of standard culture medium) for cell viability analyses. After 24 h, the culture medium was removed and 3,4-DHPAA *p*-CoA, VA and FA dissolved in standard medium culture in concentrations ranging from 0.1 µM to 100 µM were added. The controls (cells maintained in DMEM without (3,4-DHPAA *p*-CoA, VA and FA) were included on each plate. For cell cycle and apoptosis analyses, cells were treated with 10 µM and 100 µM of 3,4-DHPAA *p*-CoA, VA and FA for 24 h. For cell counting with a Neubauer chamber, cells were incubated with 1 µM and 10 µM of each compound for 24 h. For cell viability analyses cells were treated with concentrations ranging from 0.1 µM to 100 µM of each compound for 24 h.

### 4.4. Cell Viability Assays

#### 4.4.1. MTT Assay

The status of cancer cell line viability was determined by MTT (3-[4,5-dimethylthiazol-2-yl]-2,5-diphenyltetrazolium bromide; thiazolyl blue) assay (Sigma, New York, NY, USA) which substance is a pale yellow substrate that is reduced by living cells to yield a dark blue formazan product. This requires active mitochondria, and even recently dead cells do not reduce significant amounts of MTT. Exponentially growing cells were adjusted to 2.0 × 10^4^/cm^2^ with DMEM, plated in 96-well plates (Corning, Tewksbury, MA, USA) at 200 μL/well and incubated for 24 h according to the routine procedure. The cells were then incubated with 3,4-DHPAA *p*-CoA, VA and FA (0.1 µM to 100 µM) for 24 h. Each well was also incubated with MTT (10 μL/well; 5 g/mL) for 4 h. After 85 μL/well the liquid was removed and 50 μL/well sodium dodecyl sulfate was added to dissolve the solid residue. Finally, the absorbance was measured using a microplate reader (POLARIS-CELER^®^, Celer Biotecnologia, Minas Gerais, BH, Brazil) at 570 nm. The cell proliferation inhibition rate was calculated using the following formula: Cell viability (relative% of control) = (1 − average value of experimental group/average value of control group) × 100%.

#### 4.4.2. Cell Counting with Neubauer Chamber

Cells were grown to about 80% confluence in 6-well plates and treated for 24 h with 3,4-DHPAA *p*-CoA, VA and FA (1.0 µM and 10 µM). Adherent and non-adherent cells were collected and viability was assessed by mixing aliquots of cell suspensions with equal volumes of 0.4% trypan blue (GibcoBRL, Tokyo, Japan). Cells that accumulated the dye were considered dead.

### 4.5. Cell Cycle Analysis

Cells were rinsed briefly with calcium and magnesium-free phosphate-buffered saline and detached with trypsin at room temperature. After centrifugation, the cells were washed twice with phosphate-buffered saline, cells were resuspended in 1.0 mL of ice-cold Vindelov solution [49], containing 0.1% Triton X-100, 0.1% citrate buffer and 0.1 mg/mL RNase, and 50 mg/mL propidium iodide (Sigma Chemical Co., Saint Louis, MO, USA). After 15 min of incubation, the cell suspension was analyzed for DNA content by flow cytometry using a FACS Calibur flow cytometer (Becton Dickinson, Mountain View, CA, USA). The relative proportions of cells with DNA content diploid G0–G1 (2n), S phase (>2n but <4n), and G_2_/M phase (4n) were acquired and analyzed using Cell-Quest and WinMDI 2.9, respectively. The percentage of cell population at a particular phase was estimated with FlowJo software following the acquisition of 30,000 events. Cell dissociation procedure does not affect fluorescence under the experimental conditions that were used in this study or in any others of which we are aware. Nuclei of viable cells were gated according to FL-2W × FL2-A relation. Doublets and DNA fragmented nuclei were excluded from the analysis.

### 4.6. Apoptosis

To measure the rate of apoptosis, control and phenolic acids-treated cells were collected, washed twice with ice-cold PBS and resuspended in 500 µL binding buffer (BD, Pharmigen, San Diego, CA, USA). Next, 5 µL annexin V-fluorescein isothiocyanate (FITC) and 5 µL propidium iodide (PI) were added and the cells were incubated for 15 min at room temperature in the dark. FITC and PI staining was analyzed to determine the apoptotic rate. The percentage of total apoptotic cells was calculated by adding the percentages of early apoptotic gated cells (annexin V^+^/PI^−^) and late apoptotic gated cells (annexin V^+^/PI^+^). The reading was held on the flow cytometer (BD Biosciences, Franklin Lakes, NJ, USA), following the acquisition of 20,000 events on CellQuest, and the data analyzed using the FlowJo software (FlowJo v.X).

### 4.7. In Silico Approach

We used a modular toxicological predictive QSAR framework algorithm (Lazy Structure-Activity Relationships or LAZAR in silico toxicology, https://lazar.in-silico.ch/predict) [50] based on similarity of chemical alerts. LAZAR is a modular framework for predictive toxicology. Similar to the read across procedure in toxicological risk assessment, lazar creates local QSAR (quantitative structure-activity relationship) models for each compound to be predicted. Model developers can choose between a large variety of algorithms for descriptor calculation and selection, chemical similarity indices, and model building [50]. To perform the toxicological prediction in LAZAR, we designed phenolic acid chemical structure using ChemDraw and obtained the SMILE string. With the SMILE string, we predicted absorption, distribution, metabolism, excretion, and toxicological parameters (ADMET) based on QSAR similarity, using the pharmacokinetic algorithm pkCSM (http://bleoberis.bioc.cam.ac.uk/pkcsm/prediction) [51].

### 4.8. Statistical Analysis

The results presented are the mean and the corresponding standard deviation of three independent experiments performed in triplicate (*n* = 9). Data were analyzed using GraphPad Prism statistical software (version 5.04, GraphPad Software Inc., San Diego, CA, USA). The univariate analysis of variance (ANOVA) with the Tukey post-test at a 95% confidence level was used to test cell viability, cell cycle and apoptosis rate.

## 5. Conclusions

In conclusion, the data presented in this work showed that phenolic acids might play an essential role in reducing cell viability, modulating the cell cycle and controlling tumor progression through induction of apoptosis. Few studies investigated the effect of 3, 4-DHPAA, *p*-CoA, VA and FA in colon cancer cells, but what has been reported so far show an important role of these compounds as potent antioxidants and can be used in the prevention of various diseases among this cancer. 3,4-DHPAA seems to be the substance with the highest potential for in vivo studies, opening thus a series of views on the use of these compounds in the prevention and treatment of colon cancer.

## Figures and Tables

**Table 1:** Comparison between pKCSM and LAZAR toxicity prediction, and antioxidant activity of phenolic acids.

Parameters		3,4-DHPAA	*p*-CoA	VA	FA	Unit
**AMES** **toxicity**	**pkCSM**	No	No	No	No	Categorical (Yes/No)
	**LAZAR**	No	No	No	Yes	
**Hepatotoxicity**	**pkCSM**	No	No	No	No	Categorical (Yes/No)
	**LAZAR**	0.246	-		-	NA
**Oral Rat Acute Toxicity (LD50)**	**pkCSM**	1.871	1.963	2.004	1.954	Numeric (mol/kg)
	**LAZAR**	-	-	-	-	NA
**Oral Rat Chronic Toxicity (LOAEL)**	**pkCSM**	3.045	2.94	2.827	2.892	Numeric (log mg/kg_bw/day)
	**LAZAR**	-	-	-	-	NA
**Carcinogenicity (rat)**	**pkCSM**	-	-	-	_	NA
	**LAZAR**	No	No	No	No	Categorical (Yes/No)
**Carcinogenicity (mouse)**	**pkCSM**	-	-	-	_	NA
	**LAZAR**	No	No	No	No	Categorical (Yes/No)
**Carcinogenicity (rodents)**	**pkCSM**	-	-	-	_	NA
	**LAZAR**	No	No	Yes	Yes	Categorical (Yes/No)
**Max. tolerated dose (human)**	**pkCSM**	1.453	1.505	1.404	1.366	Numeric (log mg/kg/day)
	**LAZAR**	1.66	0.145	5.7	2.94	Numeric (mg/kg_bw/day)
**DPPH (25.0 µM)**		93.84	11.77	16.40	14.35	DPPH reduction (%)
**FRAP (5.0 µM)**		970.62	33.06	240.56	141.39	(μMol Ferrous Sulfate/µmol of compound)
**ABTS (5.0 µM)**		3044.75	1879.75	1376.00	2387.25	(µMol Trolox/µmol of compound)
**ORAC (3.0 µM)**		12.9	15.0	11.7	4.73	(meq Trolox/mol of compound)

NA—not applicable.

**Table 2:** Effect of 3,4-DHPAA, p-CoA, VA e FA (10.0 μM and 100.0 μM) on stages of death process in human colon adenocarcinoma cells (HT-29) after 24 h.

Compounds		Viable Cells (Annexin V − PI−)	Early Apoptosis (Annexin V + PI−)	Late Apoptosis (Annexin V + PI+)	Non-Apoptotic Cells (Annexin V − PI+)
	**CT**	89.75 ± 0.13 ^a^	3.87 ± 1.33 ^a^	3.97 ± 0.26 ^a^	0.17 ± 0.02 ^a^
**3,4-DHPAA**	**10 μM**	87.90 ± 1.04 ^b^	5.49 ± 0.28 ^b^	5.84 ± 1.48 ^a,b^	2.41 ± 2.06 ^b^
	**100 μM**	85.20 ± 0.57 ^c^	6.93 ± 1.21 ^b^	7.69 ± 0.80 ^b^	0.78 ± 0.35 ^c^
	**CT**	92.50 ± 0.28 ^a^	2.08 ± 0.23 ^a^	2.98 ± 0.04 ^a^	2.45 ± 0.47 ^a^
***p*-CoA**	**10 μM**	76.95 ± 0.64 ^b^	12.75 ± 0.49 ^b^	9.83 ± 0.16 ^b^	0.48 ± 0.06 ^b^
	**100 μM**	89.40 ± 1.48 ^c^	0.17 ± 0.10 ^c^	1.38 ± 0.24 ^c^	6.01 ± 1.11 ^c^
	**CT**	92.50 ± 0.28 ^a^	2.08 ± 0.23 ^a^	2.98 ± 0.04 ^a^	2.45 ± 0.47 ^a^
**VA**	**10 μM**	90.00 ± 4.10 ^a^	0.34 ± 0.14 ^b^	0.93 ± 0.62 ^b^	8.74 ± 3.34 ^b^
	**100 μM**	91.10 ± 2.83 ^a^	0.09 ± 0.11 ^b^	1.00 ± 0.03 ^b^	7.84 ± 2.88 ^b^
	**CT**	92.50 ± 0.28 ^a^	2.08 ± 0.23 ^a^	2.98 ± 0.04 ^a^	2.45 ± 0.47 ^a^
**FA**	**10 μM**	82.70 ± 1.27 ^b^	8.28 ± 1.53 ^b^	7.52 ± 1.68 ^b^	1.51 ± 1.10 ^b^
	**100 μM**	89.90 ± 1.84 ^c^	0.48 ± 0.07 ^a^	1.70 ± 0.18 ^a^	8.20 ± 2.28 ^c^

^a, b, c^ different letters in the same column and substance, indicate statistical difference (*p* < 0.05).

## Availability of Data and Materials

All the processed data are presented in the article. Information on raw data and materials are available from the corresponding authors upon request.
